# Effect of chronic administration of magnesium supplement (magnesium glycinate) on male albino wistar rats’ intestinal (Ileum) motility, body weight changes, food and water intake

**DOI:** 10.1016/j.heliyon.2023.e19042

**Published:** 2023-08-09

**Authors:** Ekementeabasi Aniebo Umoh, Agona Odeh Obembe, Daniel Ewa Ikpi, Offiong Ekpenyong Eniang-Esien, Joseph Okon Asuquo, Otu Otu Effiom-ekaha

**Affiliations:** a Department of Physiology, Arthur Jarvis University, Akpabuyo, Cross River State, Nigeria; b Department of Physiology, University of Calabar, Cross River State, Nigeria; c Department of Anatomy, Arthur Jarvis University, Akpabuyo, Cross River State, Nigeria

**Keywords:** Magnesium, Gastrointestinal tract, Motility, Ileum

## Abstract

Recent researches suggests magnesium as an adjuvant medication for COVID 19 patients. Magnesium relaxes skeletal muscles, an effect when prolonged in intestinal smooth muscles can cause severe discomfort such as bloating, vomiting, constipation and nausea. The objectives of this study was to ascertain if magnesium will cause relaxation of the intestinal (ileum) smooth muscles as it does in skeletal muscles. Also, this research seeks to find out the receptor pathway through which magnesium will alter motility in the gut using acetylcholine, atropine and propranolol. Ten male albino wistar rats (100–150 g) were randomly assigned into two groups (control and magnesium treated) (n = 5). Animals were acclimatized for two weeks before treatment which lasted for 6 weeks. Magnesium treated animals received oral magnesium glycinate (1600 mg/70 kg) daily while control group receive normal saline of equal volume. All animals had free access to food and water *ad libitum.* Results were analyzed at statistical level of P < 0.05. Body weight changes, food and water intake were not statistically significant. Basal contractions of ileum in magnesium treated group were significantly lower compared to control group. Propranolol significantly increased the percentage relaxation in magnesium treated group compared to the control. Atropine significantly decrease the percentage relaxation in magnesium treated group compared to the control. Higher doses of acetylcholine (10^−5^ and 10^−4^) increased the contractions in magnesium treated group. Conclusively, magnesium decreases motility of the intestine through beta adrenergic receptor pathway. Intake of magnesium for long period should be closely monitored to avoid the discomforting symptoms earlier stated.

## 1 Introduction

Magnesium shows several beneficial effect across diverse physiological systems in the body. This makes magnesium a possible supplement in the management of COVID-19 [1]. Magnesium sources include food substances and drug supplement. Magnesium glycinate, one of the several recommended supplements of magnesium is considered to be more readily absorbable in the gut and has less side effect compared to other forms of magnesium supplement [2]. In the gastrointestinal tract, magnesium supplement such as magnesium oxide is used for the management of chronic functional constipation in children [3]. Recent research has further proven that oral magnesium supplementation (800 mg magnesium oxide daily) prevents the postoperative complications of cardiac surgery, including nausea, vomiting and constipation in patients from the admission to discharge from hospital [4]. Magnesium sulfate is also used as an adjuvant analgesic drug for stomach surgery. Before sedation, intravenous magnesium sulfate at 50 mg/kg decreases analgesic requirements both during and after endoscopic submucosal dissection for gastric neoplasm without adverse effects in patients [5]. Hamedifard et al. in his research which aimed at analyzing the impacts of magnesium and zinc supplements on glycemic control, serum lipids, and biomarkers of oxidative stress and inflammation in patients suffering from coronary heart disease (CHD) and type 2 diabetes mellitus (T2DM) concluded that these two supplements had beneficial effects on fasting plasma glucose, high density lipoprotein cholesterol, C-reactive protein, insulin, total nitrite, total antioxidant capacity levels, and beck depression inventory index and beck anxiety inventory score for patients with T2DM and CHD [6]. However, the administration of magnesium supplement in these cases were mostly effective for acute treatment lasting for few days. Though administration of magnesium and zinc supplements for a period of 30 days has proven to be effective in managing type 2 diabetes mellitus and coronary heart disease [3,6] and further prolonged studies on the administration of magnesium supplement has proven to be effective in sleep disorders [7], Little or nothing is known of the adverse effect of long term administration of magnesium supplement on the gastrointestinal tract, being the first point of contact when taken orally. This research therefore seeks to close this gap in information.

Magnesium is also seen as an antagonist of calcium in skeletal muscle contraction. This makes it an essential element for skeletal muscle health as it helps to regulate the rate of skeletal muscle contraction [8]. Calcium is an important element in both skeletal and smooth muscle contractions [9]. In smooth muscles, the binding of calcium to calmodulin is necessary to effect contractions [10]. Gastrointestinal functions are mediated by smooth muscles which actually occupies a greater percentage of the gut. These functions of the gut are however controlled by nervous and hormonal mechanisms [11]. Contractions and relaxations of the gastrointestinal tract aids in diverse processes of digestion and absorption [12]. Therefore, since magnesium antagonizes calcium in skeletal muscle contractions, it will be worthwhile to assess if it will also have similar effect on calcium in smooth muscle contraction as applicable to the gut. Should magnesium antagonize calcium in gastrointestinal tract smooth muscles contractions, then many functions mediated by smooth muscles in the gut will most probably be altered.

The smooth muscles of the gastrointestinal tracts plays the motility function of the gut. There exist several disorders of the gastrointestinal tract which are associated with alterations of motility functions. These disorders includes gastroesophageal reflux disease (GERD), intestinal dysmotility or pseudo-obstruction, small intestinal bacterial overgrowth (SIBO), constipation, diarrhea, fecal incontinence, hirschsprungs's disease, gastroparesis and achalasia [13]. It is possible that the symptoms of these disorders could be seen in cases where the contractions of the gut are altered. Since magnesium antagonized calcium in skeletal muscles contraction, it is necessary to check if it will do same in smooth muscles in order to avoid or check mate the symptoms alterations in motility following the intake of magnesium. Also, since magnesium is suggested to be important in managing COVID-19 patients, this research is necessary as it will ensure that detrimental effects is not done to the gastrointestinal tract at the expense of treating COVID-19. This is because the result obtained from this research will give the clinicians more knowledge pertaining to the prescriptions of the said magnesium in any disease condition that it is found to be effective. The research will further throw more light on the effect of magnesium on food intake, water intake and body weight changes as little is known of magnesium on this aspect. Furthermore, following the use of atropine, acetylcholine and propranolol to stimulate the intestine of the rats, the research will suggest the possible mechanism of action of magnesium in exerting its effect on the gastrointestinal tract smooth muscle contractions.

## 2 Materials and methods

### 2.1 Chemicals and equipment

The chemicals used in this research and their places of purchase are as follows:

Magnesium glycinate (400 mg) purchased from Natures Lab, United States of America. Normal saline (100 ml), atropine (0.6 mg/ml), acetylcholine (20 mg), propranolol (10 mg), and methylated spirit (100 ml) were purchased from Bez Pharmacy, Calabar, Cross River State, Nigeria. Tyrode solution prepared from distilled water (800 ml), Sodium chloride (8 g), Potassium chloride (0.2 g), Calcium chloride (0.24 g), Magnesium chloride (0.1 g) and glucose (1 g) were purchased from Maxicare Pharmacy, Calabar, Cross River State, Nigeria.

The equipment/apparatus used in this research include:

Kymograph machine with tracing papers manufactured by Ningbo, Zhejiang, China. Organ bath manufactured by Adarsh International, India. Electronic weighing scale manufactured by Shanghai Dahua Scale Factory, China. Dissecting set, sowing thread, ink, and calibrated feeding bottles.

### 2.2 Preparation of stock solution of magnesium glycinate

Preparation guidelines for stock solution was used for aqueous solution of magnesium glycinate [14]. 1600 mg is the recommended dose of magnesium glycinate for a 70 kg man. Therefore, 1 g weighted animal will take 22.857143 mg of the drug. The various weight of the animals were used to calculate their individual dosage. Thereafter, the drug dose of individual animals was dissolved in 1 ml of normal saline for administration in line with Organisation for Economic Corporation and Development (OECD) guideline [14].

### 2.3 Experimental animals and design

Male albino wistar rats weighing 100–150 g were the animals of choice for this research. Animals were acclimatized for two weeks and separated into two groups (Control and Magnesium treated) for administration. Animals in magnesium treated group received stock solution of magnesium glycinate at 22.84 mg/kg body weigh while those in Control group received equal volume of normal saline for a period of 6 weeks. All animals were placed in separate metabolic cages and allowed free access to food and water throughout the period of administration in the animal facility of Arthur Jarvis University.

### 2.4 Determination of body weight changes, food and water intake

Daily body weight of the animals were taken throughout the period of the research using a weighing balance. Body weight changes for each group were obtained by subtracting the initial body weight of each animal at the start of administration from the final body weight at the end of administration.

Water intake was measured using calibrated feeding bottle with stainless steel nozzles. The volume of water consumed per day for each animal was obtained by subtracting the volume of water remaining at the end of 24 h of feeding from the initial amount in the feeding bottle at start of the day. The result gotten throughout the period of administration was added to obtain the total water intake of each animal per group.

The food intake was measured by weighing the amount of food left in the container after 24 h and subtracting it from the initial amount of food at the start of the day's feeding. The result obtained throughout the period of administration was added to obtain the total food intake of each animal per group.

### 2.5 Determination of intestinal motility

Determination of intestinal motility was done using a kymograph machine with an organ bath. The rats were starved for 24 h to ensure complete absent of food in the intestine before the experiment. The animals were sacrificed by stunning and an incision was quickly made through the linea alba to expose the intestine. The proximal ileum was isolated, placed in a container of Tyrode solution and aerated. Thereafter, the ileum was cut into segment of about 3 cm long and mounted at one end to a fixed support in an organ bath. The other end of the ileum was fixed to a horizontal balance writing lever tangential to the kymograph drum. The tissue were allowed to equilibrate for 60 min. During the period, the bathing solution was replaced with Tyrode solution at 15 min interval to avoid accumulation of metabolites and the record on the kymograph tracing paper was duly noted. The tissue (ileum) in the organ bath was later challenged with graded doses of acetylcholine (10^−4^, 10^−5^, 10^−6^, 10^−7^, 10^−8^ and 10^−9^) and the tracings on the kymograph paper by the writing lever was duly noted. The Tyrode solution was flushed and replaced with same volume of newly prepared ones. Thereafter, atropine (0.1 ml) was administered on the tissues. The effect of atropine as recorded on the tracing paper by the writing lever was duly noted for a period of 15 min. Thereafter, the tyrode solution was replaced again and propranolol (0.1 ml) was further administered to see the response of the tissues as recorded on the tracing paper. At the end of the experimental procedures, positive waves on the tracing paper which strokes upward indicated contractions whereas negative waves which strokes downward indicated relaxation of the tissues. These waves on the tracing paper were used to analyze the motility of the intestinal smooth muscles at basal levels and subsequent administration of acetylcholine, atropine and propranolol.

### 2.6 Ethics approval and consent to participate

All experiment were performed in accordance with the guideline for care and use of laboratory animal of the Faculty of Basic Medical Science Animal Research Ethics Committee (FAREC-FBMS), Arthur Jarvis University.

### 2.7 Statistical analysis

All results were presented as mean ± standard error of mean (SEM). Student independent T test was used for the analysis of the data. A computer software (Microsoft excel, 2018 version) was used for the analysis of the result. The results were considered significant at the level of P < 0.05.

## 3 Results

### 3.1 Total food intake among experimental groups

**Fig. 1:**
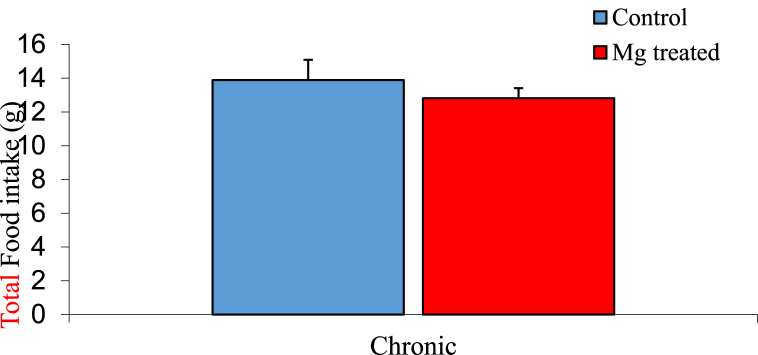
Graph showing total food intake of the control and magnesium treated groups. n = 5. values are not statiscally significant.

The mean ± SEM daily food intake of the control and magnesium treated group 13.89 ± 1.20 g and 12.82 ± 0.59 g respectively. The result showed no significant difference at P < 0.05 between the control and the magnesium treated group as shown in Fig. 1.

### 3.2 Total water intake among experimental groups

**Fig. 2:**
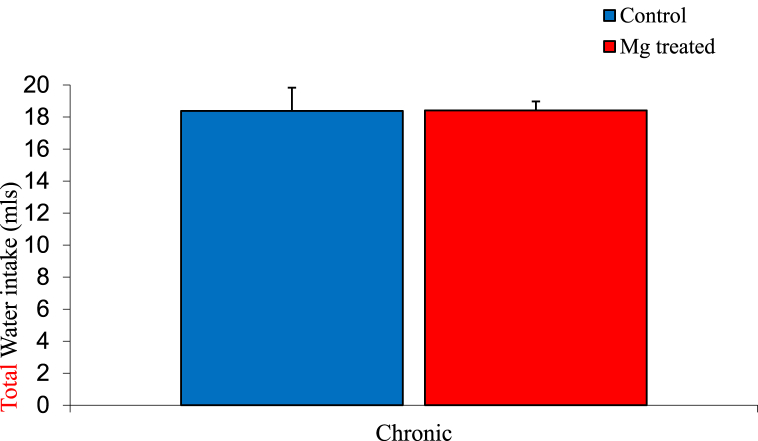
Graph showing water intake of the control and magnesium treated groups. n = 5Values are not statiscally significant.

The mean ± SEM of total water intake of the control and magnesium treated group were 18.83 ± 1.45 mls and 18.41 ± 0.56 mls respectively. The result showed no significant difference between the control and magnesium treated group as shown below in Fig. 2.

### 3.3 Body weight changes between experimental groups

**Fig. 3:**
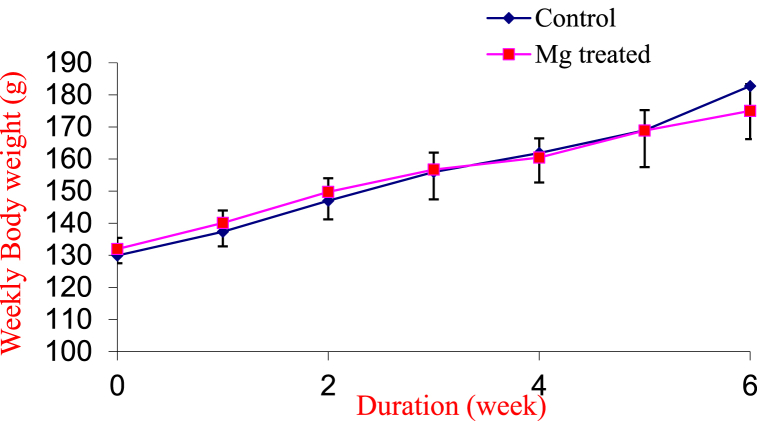
Weekly body weights of the control and magnesium treated groups. Values are expressed as Mean SEM. n = 5.

The mean ± SEM body weight changes at the end of the experimental period of the control and magnesium treated group were 52.82 ± 14.71 g and 42.06 ± 6.38 g respectively. The result showed no significant difference between the experimental groups as shown in Fig. 3.

### 3.4 Basal height contractions of experimental groups

**Fig. 4:**
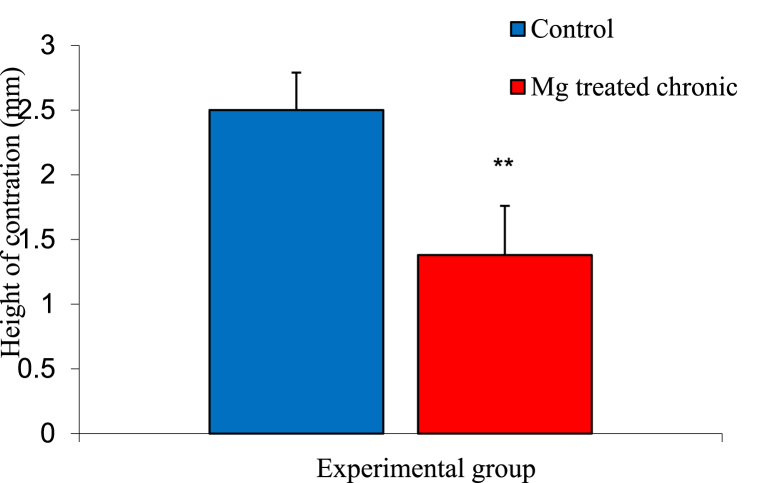
Graph showing basal height of contraction in the intestinal smooth muscle of control and magnesium treated groups. n = 5. ** = p < 0.05 vs Control.

The mean ± SEM of basal ileal height of contractions in the control and magnesium treated groups were 1.48 ± 0.02 mm and 2.50 ± 0.01 mm respectively. The analysis of result showed a significant decreased in the magnesium treated group compared to control as shown in Fig. 4.

### 3.5 Effect of acetylcholine on contractions of experimental groups

**Fig. 5:**
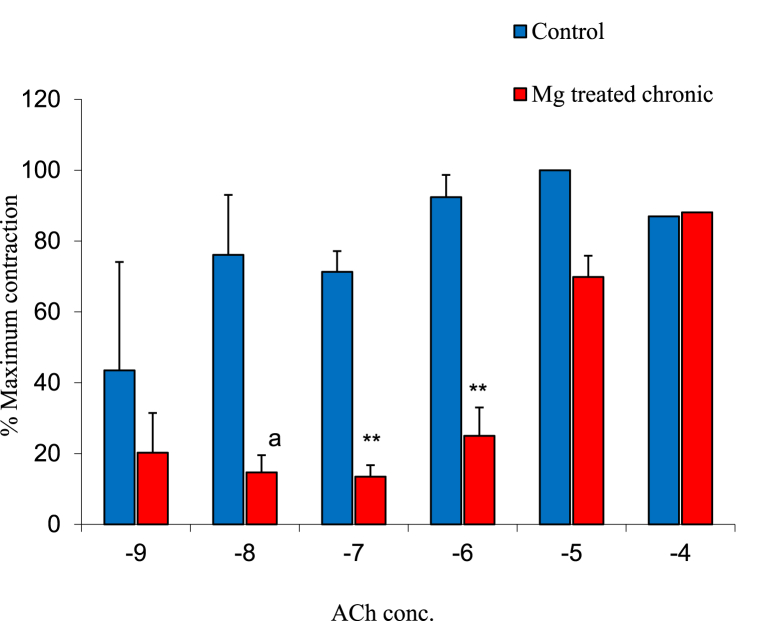
Graph showing percentage maximum contraction of the ileal smooth muscle to graded concentrations of ACh in the control and magnesium treated groups. n = 5. ** = p < 0.05 vs Control.

The mean ± SEM of percentage maximum contraction of the ileal smooth muscle to acetylcholine (Ach) concentrations of 10^−9^, 10^−8^, 10^−7^, 10^−6^, 10^−5^ and 10^−4^ in the control and magnesium treated group were 33.33 ± 30.06% and 20.24 ± 11.23%, 60.00 ± 16.74% and 14.68 ± 4.87%, 76.09 ± 5.86% and 13.49 ± 3.21%, 92.39 ± 6.28% and 25.00 ± 8.03%, 100.00 ± 12.55% and 88.10 ± 6.94% as well as 80.96 ± 20.08% and 88.10 ± 6.94% respectively. The result showed a significant decrease in ileal muscle contraction of the gastrointestinal tract at 10^−7^ and 10^−6^ Ach concentration. However, higher doses of 10^−5^ and 10^−4^ acetylcholine concentration increased the contractions in magnesium treated group as shown in Fig. 5.

### 3.6 Effect of atropine on ileal muscle contraction of experimental groups

**Fig. 6:**
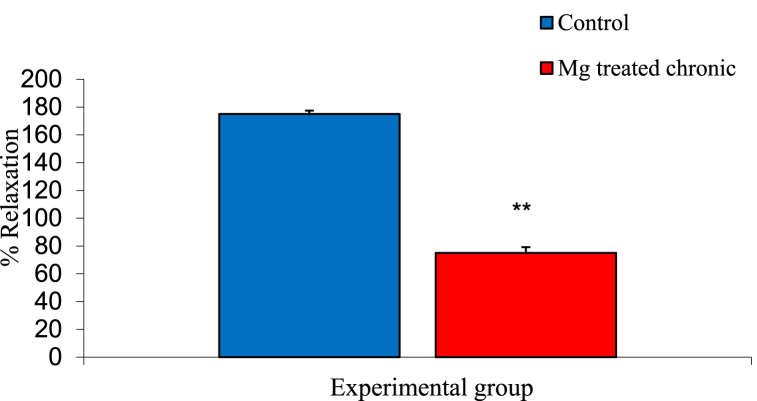
Graph showing effect of atropine on ileal motility in control and magnesium treated groups. n = 5. ** = p < 0.05 vs Control.

The mean ± SEM percentage relaxation of the intestinal smooth muscles of control and magnesium treated animals following administration of 0.1 ml atropine were 175.00 ± 14.43% and 75.00 ± 14.43% respectively. The result showed a significant decreased in magnesium treated group compared to the control group as shown in Fig. 6.

### 3.7 Effect of propranolol on ileal smooth muscle contraction of experimental groups

**Fig. 7:**
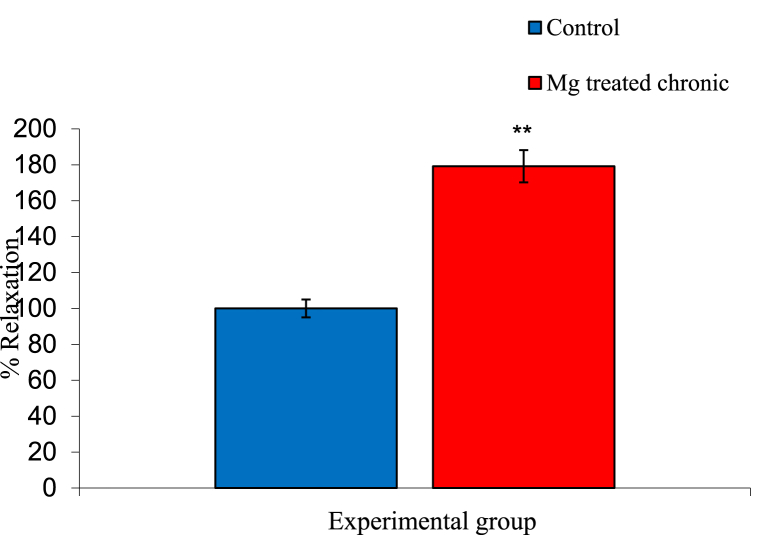
Graph showing effect of propranolol on ileal motility in control and magnesium treated group n = 5. ** = p < 0.05 vs Control.

The mean ± SEM percentage relaxation of ileal smooth muscle of control and magnesium treated group following administration of 0.1 ml of propranolol were 216.67 ± 9.62% and 100.00 ± 0.00% respectively. The result showed a significant increase in relaxation on magnesium treated group animals compared to the control group as shown in Fig. 7.

## 4 Discussion

Magnesium as an enzymatic activator is essential for various physiological functions such as cell cycle, metabolic regulation, muscle contraction and vasomotor tone. Magnesium and Zinc supplement are vital in managing type 2 diabetes mellitus, a disease condition associated with the pancrease and gastrointestinal tract as a whole [6]. Also, A growing body of evidence supports that magnesium supplementation (mainly magnesium sulfate and magnesium oxide) prevents or treats various types of disorder or diseases related to respiratory system, reproductive system, digestive system, nervous system and cardiovascular system as well as kidney injury, diabetes and cancer [1]. Functions of the gastrointestinal tract can be altered due to the intake of certain food substances, herbs or drugs. At same time, herbs, drugs and certain food substances might also be a form of remedy for this altered functions. For instance, opioids and ginger has been found to be beneficial in the treatment of inflammatory bowel disease, a subgroup of persistent, long term, progressive and relapsing inflammatory conditions that is most applicable in the colon of the gastrointestinal tract [15,16]. Also, ginger has been considered an anti-inflammatory medicinal herb which has tendencies to increase pepsin secretion, decrease mucus content and subsequently raise the ulcer scores of rat's stomach when compared to the control group [17]. Such could be the case of magnesium as it might be beneficial or detrimental to the gastrointestinal tract at the expense of using it to treat disorders of other physiological systems in the body. The benefits of magnesium has also been put forward by some authors, however some of these benefits were demonstrated following a combined administration of magnesium with other supplement such as zinc in diabetes management as put forward by Hamedifard et al., in 2020 [6]. The most recent suggestion of magnesium in managing COVID-19 as put forward by Tang et al. [1] makes it necessary to explore the effect of magnesium on other aspect of the gastrointestinal tract, a system that makes the first contact with magnesium following oral administration.

In the gut, magnesium has been seen to be effective in treatment of constipation, diarrhea, nausea, post-surgical pains and even diabetes mellitus (type 2) [3,4,6]. However, these treatment were effective for acute periods lasting a few days or about one to two weeks. There exist cases where magnesium has been used continuously for over long periods as seen in sleep disorders, sustained eclampsia, Covid-19 and even diabetes [1,7,6]. Little or nothing is known of the adverse effect of this supplement in the gastrointestinal tract over these long period of administration. Hence complications following such administration may not be properly handled and treated. Most complications in the gastrointestinal tract are usually linked to various movements that occurs in the gut. For instance, achalasia, gastroesophageal reflux disease (GERD), gastroparesis and even biliary dyskinesia are all due to disorders of motility [9]. Gastroparesis which is characterized by delayed gastric emptying without evidence of mechanical obstruction as a result of decreased motility can cause nausea, vomiting, early satiety and abdominal pains. Magnesium which often lead to relaxation in skeletal muscle was also seen to decrease intestinal smooth muscles in this research. Hence, the symptoms seen in decreased motility of the gut will as well be applicable in chronic usage of magnesium. There is also no doubt that this relaxation of the intestinal smooth muscle caused by magnesium can as well lead to possible symptoms of gastroparesis such as nausea, vomiting, early satiety and even abdominal pains over long period of administration. Therefore, although magnesium might be beneficial in handling constipation, vomiting and nausea at acute administration, its long term effect of relaxing the smooth muscles will subsequently result in the same set of symptoms it was said to alleviate earlier on.

The mechanism through which magnesium causes relaxation is essential as it will give clinicians a better method of administering magnesium to patients. Aside, relaxation effect, magnesium acts through various mechanism to mediates its function. If the adverse effect seen in long term administration of magnesium is due to its relaxing capabilities on intestinal smooth muscles, then subsequent blocking of the relaxation pathway mediated by magnesium will compensate for this adverse effect and enhance other intended functions of magnesium as prescribed by clinicians. In this research, basal heights of contraction was decreased in magnesium treated group compared to the control. This result was in line with that of Zocchi et al. [8] as seen in skeletal muscles. It further goes in line with other research which shows magnesium to be a relaxant of arterial smooth muscle [18]. Also, administration of acetylcholine showed subsequent increase in contractions of magnesium at higher doses of acetylcholine. However, the increase contractions were still lower than contractions seen in control group. This finding was also in line with study of Altura et al. [19] where it was shown that of all calcium antagonist, only magnesium has the capability to inhibit myogenic, basal and hormonal-induced vascular tone of muscular contraction. Acetylcholine binds to muscarinic receptors (a G-protein couple receptor) in muscles to cause contraction [20]. Atropine, on the other hand is considered blockers of acetylcholine. It acts as a competitive, reversible antagonist of muscarinic receptor: an anticholinergic drug [21]. Atropine decreased relaxation (increase contraction) in magnesium treated group ileal smooth muscles. This result suggest that the mechanism of relaxation caused by magnesium on the ileal smooth muscle does not follow the cholinergic system or muscarinic receptor pathway. However, administration of propranolol showed a significant increase in relaxation of the magnesium treated group compared to the control. Propranolol which is a competitive beta-adrenergic receptor antagonist devoid of agonist activity, is often used as a prototype for comparison to other beta-antagonist [22]. It exerts its response by competitively blocking beta-1 and beta-2 adrenergic stimulation typically induced by epinephrine and norepinephrine [23]. Therefore, in this research, it was evident that aside antagonizing calcium, a possible mechanism through which magnesium mediates it relaxation effect is in a way similar to that of propranolol via binding to beta-adrenergic receptors in the gastrointestinal tract.

## 5 Conclusion

In light of this study, it can be concluded that magnesium exerts a relaxation effect on intestinal (ileal) motility by binding to beta adrenergic receptors in same manner as propranolol. Following the relaxing effect of magnesium, there will likely be symptoms of pains, nausea, vomiting and even constipation at chronic administration, as against its ameliorating property of this same symptoms at acute administration. A possible approach to administering magnesium for longer periods is giving it with an adjuvant medication that can block its pathway of relaxation. By so doing, magnesium may not be able to mediate its relaxation effect but will however carry out its other functions as intended by clinicians.

## 6 Limitation of the study

This study is limited by small sample size and further research is needed to confirm and extend these findings, and to investigate the clinical implications of magnesium's effects on intestinal motility.

## Ethics approval and consent to participate

All experiment were performed in accordance with the guideline for care and use of laboratory animal of the Faculty of Basic Medical Science Animal Research Ethics Committee (FAREC-FBMS), Arthur Jarvis University.

## Author contribution statement

Ekementeabasi Aniebo Umoh: Conceived and designed the experiment.

Obembe Agona Odeh: Performed the experiment.

Ikpi Daniel Ewa: Analyzed and interpreted the data.

Offiong Ekpenyong Eniang-Esien, Joseph Edet Asuquo and Effiom-ekaha Otu Otu: Contributed reagents, materials, analysis tools or data.

Ekementeabasi Aniebo Umoh: Wrote the paper.

## Funding statement

This research did not receive any specific grant from funding agencies in the public, commercial, or not-for-profit section.

## Additional information

No additional information is available for this paper.

## Declaration of competing interest

The authors declare that they have no known competing financial interests or personal relationships that could have appeared to influence the work reported in this paper.
